# The Reasons for the Low Uptake of New Antidiabetic Drugs with Cardiovascular Effects—A Family Doctor Perspective

**DOI:** 10.3390/jcm13061617

**Published:** 2024-03-12

**Authors:** Tomislav Kurevija, Dunja Šojat, Zvonimir Bosnić, Blerim Mujaj, Silvija Canecki Varžić, Ljiljana Majnarić Trtica

**Affiliations:** 1 Department of Family Medicine, Faculty of Medicine, Josip Juraj Strossmayer University of Osijek, J. Huttlera 4, 31000 Osijek, Croatia; tkurevija@mefos.hr (T.K.); dsojat@mefos.com (D.Š.); zbosnic191@gmail.com (Z.B.); 2 Research Association Alliance Institute for the Promotion of Preventive Medicine (APPREMED), 2800 Mechelen, Belgium; 3 General Practice, Huisartsenpraktijk, Bremtstraat 116, 9320 Aalst, Belgium; 4 Department of Pathophysiology, Faculty of Medicine, Josip Juraj Strossmayer University of Osijek, J. Huttlera 4, 31000 Osijek, Croatia; silvija.canecki@mefos.hr; 5 The Clinic for Internal Disease, Unit for Endocrinology and Diabetes, Clinical Hospital Centre Osijek, J. Huttlera 4, 31000 Osijek, Croatia

**Keywords:** type 2 diabetes, therapeutic inertia, clinical guidelines, sodium-glucose cotransporter-2 inhibitors, glucagon-like peptide-1 receptor agonists, primary healthcare

## Abstract

Chronic diseases, such as type 2 diabetes (T2D), are difficult to manage because they demand continuous therapeutic review and monitoring. Beyond achieving the target HbA1c, new guidelines for the therapy of T2D have been introduced with the new groups of antidiabetics, glucagon-like peptide-1 receptor agonists (GLP-1ra) and sodium-glucose cotransporter-2 inhibitors (SGLT2-in). Despite new guidelines, clinical inertia, which can be caused by physicians, patients or the healthcare system, results in T2D not being effectively managed. This opinion paper explores the shift in T2D treatment, challenging assumptions and evidence-based recommendations, particularly for family physicians, considering the patient’s overall situation in decision-making. We looked for the possible reasons for clinical inertia and the poor application of guidelines in the management of T2D. Guidelines for antidiabetic drugs should be more precise, providing case studies and clinical examples to define clinical contexts and contraindications. Knowledge communication can improve confidence and should include clear statements on areas of decision-making not supported by evidence. Precision medicine initiatives in diabetes aim to identify subcategories of T2D patients (including frail patients) using clustering techniques from data science applications, focusing on CV and poor treatment outcomes. Clear, unconditional recommendations for personalized T2D management may encourage drug prescription, especially for family physicians dealing with diverse patient contexts and clinical settings.

## 1. Introduction

Managing chronic diseases is challenging, as they require continuous monitoring and the evaluation of therapy. For this reason, chronic diseases are usually not well controlled in clinical practice [1]. The guidelines for managing these diseases are rapidly changing, which also contributes to clinical inertia—the healthcare professionals’ inability to initiate or intensify therapy when desired therapeutic goals are not achieved. Clinical inertia is considered a kind of medical error that can adversely affect health-related outcomes and contribute to an increase in healthcare costs [2]. 

Factors that contribute to clinical inertia can be attributed to doctors, patients or the healthcare system, but they are often interconnected [2,3,4] (Table 1). In a narrow sense, clinical inertia refers to the poor adherence of healthcare providers to evidence-based recommendations for medication therapy, which is termed “therapeutic inertia” [4,5]. It is important to make this distinction because it helps differentiate doctor-related causes of clinical inertia from patient non-adherence to pharmacological treatment [6].

Type 2 diabetes (T2D) is a common chronic disease, especially among the older population, and its prevalence is expected to increase in the years that come due to the epidemic of obesity and global population aging [7]. Clinical inertia and a low adherence to evidence-based recommendations are common issues in managing T2D [8,9]. There are many reasons for that. Apart from well-known cardiovascular (CV) complications, T2D is also associated with a higher risk of age-related conditions such as sarcopenia, malnutrition, falls, urinary incontinence and cognitive impairment, which makes it one of the most disabled diseases [10]. Multiple comorbidities and complicated care regimens significantly affect the quality of life of these patients. Consequently, healthcare providers face a great challenge in delivering quality care for these patients [11]. Especially, family physicians are under immense pressure due to their role of providing comprehensive and patient-centered care [12]. 

With the emergence of new groups of antidiabetic drugs, such as glucagon-like peptide 1 receptor agonists (GLP-1ra) and sodium-glucose cotransporter-2 inhibitors (SGLT2-in), which have significant cardio- and renal-protective effects, the pharmacological treatment of T2D has begun to undergo revolutionary changes [13]. With the appearance of these drugs in the market, the programmed request, outlined in the international actionable documents to more efficiently combat cardiovascular disease (CVD), has come to be realized [14]. The possibility for the integrated management of T2D and CVD would be of the utmost importance for public health efforts because of the global epidemic of T2D and the fact that CVD is a leading cause of death worldwide. This is even more so considering that these conditions share common risk factors and pathophysiology pathways [15,16]. However, despite the proven efficacy of novel antidiabetic drugs in reducing CV morbidity and mortality in T2D patients, their prescription rates remain low in many countries and across clinical disciplines [17]. 

In this opinion paper, we will explore the issues mentioned above, discussing the challenges in managing chronic diseases like T2D, highlighting the introduction of new guidelines for managing T2D and new antidiabetic drugs, addressing clinical inertia and advocating for precision medicine initiatives for improving the personalized management of T2D patients, particularly emphasizing the role of family physicians in decision-making and patient care. This critical viewpoint refers primarily to the common guidelines of the American Diabetes Association (ADA) and the European Association for the Study of Diabetes (EASD) and their recent updates, as widely used among providers in European countries [18,19,20]. We will also illustrate some of our critiques using data collected through our research. 

## 2. Hesitancy in Delivering a New Antidiabetic Drug Treatment Strategy Related to Postulates of Clinical Inertia

To determine the extent of clinical inertia, three postulates are crucial: (1) the specific clinical outcomes that the treatment is intended to achieve, (2) the recommended therapy regimen that should be administered and (3) the timeframe for the timely intensification of therapy [2]. Regarding the first postulate, intermediate biomarkers, which measure the quality of care in T2D patients, like hemoglobin A1c (HbA1c), blood pressure and low-density lipoprotein (LDL)-cholesterol, have been preferred in most studies over end-point clinical outcomes, like general or CV mortality, or hospital admission rates, as the latter requires long-term follow-up [5,9]. The laboratory marker, HbA1c, has been traditionally used as a standard to guide medication therapy in clinical guidelines and practice [13]. The development of novel anti-diabetic medications with CV advantages caused a change in treatment goals, with the main objective now being to reduce CV risk rather than just controlling glucose [18,20]. Healthcare providers might have faced a problem while making decisions regarding this switch. Even more so, the current ADA/EASD guidelines still recommend achieving optimal glycemic control to minimize the risk of cardiac events in the long term [19,21]. Therefore, there is a need to meet the dual goal. 

The question being considered is whether combining a classical hypoglycemic drug with a new-class drug, when optimal HbA1c is not achieved, is beneficial in the long run or if the episodes of hypoglycemia, as evidence suggested, may cause harm, also regarding long-term health outcomes [22]. It might be a dilemma, especially in older T2D patients with multiple health conditions, who are, however, the right candidates for new-class drugs [18,21]. On the other hand, does maintaining a suboptimal HbA1c diminish the positive CV effect of the new-class drug, in any manner, to avoid hypoglycemia? Two lines of concern that healthcare providers face are evident in this dilemma. The first one is the fear of over-treating and resulting hypoglycemia—a known reason for clinical inertia when managing T2D patients [23,24]. The second is the uncertainty associated with long-term outcomes, which is difficult to predict in individual patients, as multiple factors that characterize patients, and variations in treatment, can influence them [25]. The providers’ hesitancy to intensify or change the therapy is not always due to indolence but rather the fear of harming or the difficulty of deciding conditions of uncertainty. The abovementioned is supported by studies demonstrating that therapeutic inertia increases with the prescription of more antidiabetic drugs over time and decreases when HbA1c levels rise [5]. 

Regarding the time from the problem detection to therapy intensification, the studies that were conducted before the advent of new antidiabetic drugs reported a long time delay despite the suboptimal glycemic control [26,27]. How is one to know whether some of the GLP-1ra or SGLT2-in is delivered timely when there is more than one criterion to follow? Is early therapy intensification the right choice in treating newly diagnosed, usually obese, T2D patients, and do they need to be treated with new antidiabetic drugs immediately or when clear signs of heart failure or renal function impairment become visible? How these choices fit into the national health strategy in terms of financial restraints is also crucial. However, the intensification of therapy for newly diagnosed T2D patients has always been a challenge [28]. The 2018 update of ADA/EASD guidelines provides an alternative prescribing policy for low-income countries, which is not based on evidence-based recommendations [18]. Sharing the results of cost-effectiveness studies on new antidiabetic drugs with national healthcare authorities could help overcome therapeutic inertia [29]. 

The primary healthcare system’s ability to systemically screen T2D patients with low renal function—information that is crucial for evaluating T2D patients with chronic kidney disease (CKD)—remains an obstacle, particularly for family physicians. In particular, to obtain information on quantified heart failure (whether it is with preserved or reduced ejection fraction), an ultrasound examination is required, making it unreliable to assume that all T2D patients have this information. The recent trials, examining the structured model of care—that is, the model with standardized elements of the care process—have shown benefits for quality of care, clinical outcomes and cost-effectiveness [30,31]. 

**Figure 1:**
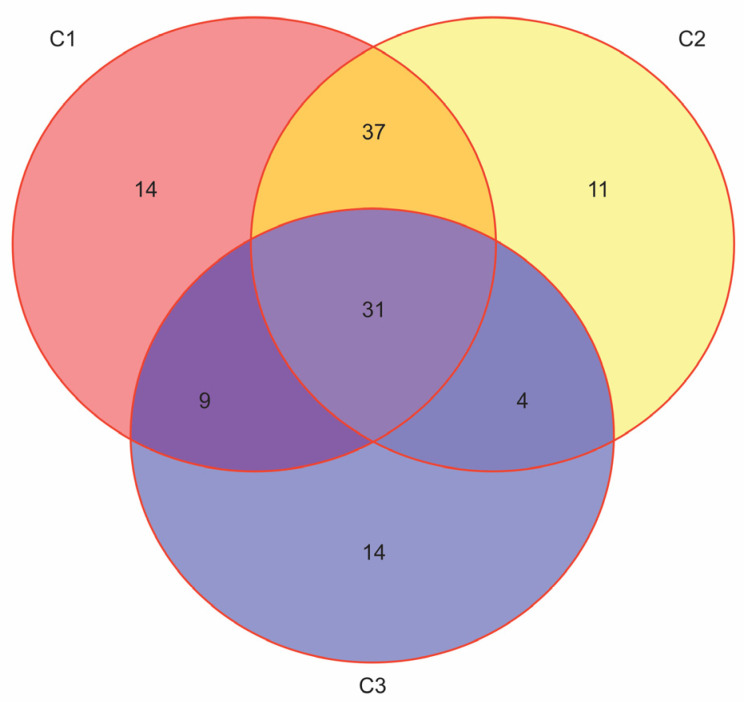
The overlap rates among the eligible patients for treatment with GLP-1ra or SGLT2inh. The unpublished results from our research that is cited in refs. [32,33] (N = 170, F:M = 95:75, age 50–89 years, median 66). The simplified criteria for prescribing GLP-1ra or SGLT2inh: C1: Atherosclerotic CVD (Coronary artery disease, Periphery artery disease or Cerebrovascular disease); C2: Age ≥ 55 years + chronic heart disease; C3: estimated glomerular filtration rate ((eGFR) < 60 mL/min).

Finally, considering that some patients may meet more than one set of indication criteria, how can the proportion of T2D patients who qualify for therapy with novel antidiabetic drugs be determined? (Figure 1). 

The clinical guidelines offer recommendations for delivering standardized and optimal care to patients based on scientific evidence. Adherence to guidelines by healthcare providers is considered the principal way to limit clinical inertia [4]. Despite the proven benefits, implementation rates remain below 50% historically across clinical disciplines [4,34]. Many attempts have been made to identify barriers that limit the implementation of clinical guidelines in daily medical practice [35,36]. It has been found that many guideline manuals are too long and complicated, making it difficult to implement them effectively. Furthermore, they often fail to offer solutions for specific patient scenarios. Research on implementation strategies for guidelines needs to be intensified. Implementing guidelines should be part of a wider process of knowledge translation that results in the creation of protocols and workflows. Given the abundance of guidelines available, there is a growing need for continuous quality assessment and the improvement of the development process [8,34,35]. In today’s era of healthcare system digitalization, online tools such as websites, alerting systems, interactive platforms and clinical decision support systems are being used to aid in the access and implementation of clinical guidelines [36,37,38]. However, the effects on practice and clinical outcomes from these tools are still unknown. 

In the diagnosis, treatment and follow-up of chronic diseases, primary healthcare providers, mostly family physicians, play a central role [3]. They are even more concerned than specialist endocrinologists with decisions on the right time to change or intensify therapy in T2D patients because their decision-making is more complex and involves the care of the whole patient [39]. This may be particularly challenging in circumstances such as starting insulin therapy, introducing new medications in the treatment plan or attempting to achieve glycemic control by combining two or more oral antidiabetic drugs, as is usual in older patients with multiple comorbidities [40]. In such cases, family physicians tend to pass the responsibility of choosing the treatment to endocrinologists, trying to avoid any anxiety that may arise due to the side effects of intensifying the therapy or using medications that they have less experience with [41]. 

It can be challenging to implement clinical guidelines in primary care and family medicine due to the specific nature of the workflow, which involves a holistic approach to patient care by considering patient preferences for treatment and their ability to take medications independently and predicting their adherence to suggested treatment [25]. It is assumed that education on psychological factors that influence patient behavior in care uptake would reduce the providers’ uncertainties during decision-making and should become a part of implementation strategies [42]. In addition, family physicians may have strong attitudes towards certain medications that they have experience with and that are well accepted by patients and may be hesitant to try new treatments, despite the evidence of their efficacy [41]. Based on that, it is important to research the psychological factors that may influence family doctors’ attitudes towards their behavioral intentions and decisions for action, as well as doctor–patient communication, which can shape behavioral changes. This research should be an integral part of the process of clinical guidelines’ development [43]. 

Overall, clinical guidelines are generally not designed to suit the working style of primary care providers and family physicians [39,44]. In addition, there is a lack of competency-based workflow and effective communication models between different disciplines, which can also contribute to the non-adherence of family physicians to the guidelines [41]. The research performed thus far has identified several categories of barriers and enablers regarding the implementation of clinical guidelines in primary care [8,45,46]. Suboptimal inter-professional communication, the non-transparent division of professional responsibilities, time constraints, the limited applicability of the guidelines in real-life practice, the lack of knowledge and skills of primary care providers (the lack of implementation strategies), a poor motivation to use guidelines in everyday workouts and inadequate reinforcement were among the most commonly reported barriers. The most frequently reported enablers were the presence of technical support, timely education and training for both primary care providers and patients. It is widely assumed that conducting qualitative research among primary care providers and family physicians could be a valuable way of gathering information on guideline gaps and barriers in implementation strategies that concern them specifically [46]. 

## 3. Trends in the ADA/EASD Guidelines on the T2D Patient Management Strategy and the Effect on New Antidiabetic Drug Uptake

The recent updates of the ADA/EASD guidelines (2018, 2019 and 2022), and the position statements drawn from them, have brought several areas of improvement for managing T2D patients [18,19,20,47]. First, the requirement is emphasized for the systematic screening of T2D patients for CVD and increased CV risk and the treatment of these patients with antidiabetic drugs with proven CV efficacy. It marked the end of the long-standing paradigm that T2D is the risk equivalent of coronary artery disease (CAD), or, in other words, that all patients diagnosed with T2D have the same risk for atherosclerotic CVD (ASCVD). The studies conducted in the past two decades have revealed that risk levels in T2D patients approach the CAD risk levels after a decade of T2D duration, in patients with target organ damage (proteinuria, estimated glomerular filtration rate < 30 mL/min/1.73 m^2^, left ventricular hypertrophy or retinopathy) or in those with three or more CV risk factors [48]. This finding allowed for the grading of CV risk in T2D patients in terms of three categories: very high, high and moderate levels. The target management of those at high and very high CV risk levels has become possible thanks to the results of CV outcome trials (CVOTs) that have become mandatory for all novel drugs used to treat T2D patients [49]. 

To date, significant CV benefits have been proven for several GLP-1ra and SGLT2-in drugs, and they are advised as a preferred treatment choice for patients with T2D and established ASCVD or those at an increased risk for ASCVD, independently of the glucose control level and the background use of metformin (the first-line medication). The therapy with SGLT2-in takes precedence over GLP-1ra for T2D patients with chronic heart failure (CHF) and chronic kidney disease (CKD) [21,49]. In addition, an increased CV risk can be ameliorated through the potent effect of these drugs—in particular, GLP-1ra and the emerging insulin secretion stimulating drug tirzepatide—on body weight reduction [50,51]. 

Nevertheless, the uptake rates of GLP-1ra and SGLT2-in drugs remain low. The potential barriers in their prescription, which refer to the gaps in the guidelines, include safety concerns, a lack of clarity about when the right time is and to whom, exactly, the therapy with either of these drugs should be initiated as well as the lack of understanding of the treatment effects such as glycemic, cardiac and renal outcomes, including possible side-effects, concerning the specific sociodemographic, clinical and laboratory characteristics of patients to whom these drugs are to be prescribed [52,53,54]. For example, differences in patient socioeconomic status may be important for the uptake of SGLT2-in if the costs are a concern [52]. A lesser glycemic response can be expected for SGLT2-in in cases of lower renal function and for GLP1-ra in cases of a patient’s reduced insulin secretion ability [53]. The knowledge is still insufficient on the full range of health effects of new antidiabetic drugs (in particular, SGLT2-in), and safety issues cannot be predicted with sufficient confidence [55,56,57]. An introduction of these drugs into the existent therapeutic scheme, particularly for older patients with multiple comorbidities and polypharmacy, may potentially cause interactions with other treatments or disease-related conditions, which may cause concerns among providers and family physicians about indications for these medications’ therapy [53,58,59,60] (Table 2).

The question that needs to be clarified is how much variation between drugs, trial designs or patient characteristics may influence the differences in the treatment effects of these drugs. In trial designs, significant differences between CVOTs have been detected. Post hoc analyses, with patient re-grouping into different CV risk categories, have been suggested as a solution to overcome these disparities [54]. In addition, the structure of patients included in CVOTs does not match the characteristics of T2D patients in real-life conditions, which could undermine the providers’ confidence in recommendations provided by the guidelines [61]. Recognizing this problem, the CVOT Summit 2022 has issued a Report with recommendations for analyzing the real-world data (that are routinely collected in practice) in order to complement the results of CVOTs by providing more specific information on treatment options, patient risks and safety issues related to these medications, which could help the decision-making process [62]. For example, an insight into the safety of SGLT2-in in specific patient groups such as elderly individuals has been obtained in this way [63]. 

The general expert view is that the guidelines should be more precise in terms of how to use these drugs in specific situations by providing case studies and clinical examples to more specifically define clinical contexts in which to initiate their use or for which contraindications may exist [52]. Similarly, family physicians assume that their confidence can be improved by strategies such as appropriate knowledge communication with them. They also assume that the guidelines should contain clear statements about which areas of decision-making are not sufficiently supported by evidence and for which subgroup of patients the harm–benefit ratio is not well understood when using new antidiabetic drugs [46,64]. 

Many of these gaps, which can lead to difficulties in making decisions about individualized therapy for T2D patients, are the result of a lack of study data. Nevertheless, in the development of the recent updates to the ADA/EASD guidelines for the management of T2D patients, there is a clear intent to promote a patient-centered approach that could help healthcare providers, particularly family physicians, alleviate concerns in decision-making and ultimately reduce clinical inertia [18,19,20]. According to these updated guidelines, the choice of antidiabetic drugs should be made with the patient in mind. Consideration should be given to comorbidities but also to the risk of hypoglycemia, the impact on weight, the treatment cost, the side effects and the patient’s preferences. In addition, the 2022 ADA/EASD guidelines focus on the social determinants of health and healthcare systems and provide the necessary evidence for counseling patients on non-pharmacological treatment and behavior changes, including physical activity, sleep hygiene, weight management and nutrition [19]. While the ADA/EASD guidelines still do not explain the need for the systematic assessment of mental health and well-being in T2D patients, the openness for continued knowledge development and implementation into practice is clearly outlined. 

## 4. T2D Patient Complexity and Endeavor towards Precision Medicine

The possibilities of the medication treatment of T2D patients are expanding, and advances in technology support such as the continuous or intermittent scanning of glucose levels, mobile health, digital support and visualization systems are leading to improvement in the management of these patients [18,19,20]. On the other hand, the requirements for a high standard of care, which include patient-centered and personalized approaches, may be constrained by q still insufficient understanding of patient complexity. The complexity means a great variability among patients in clinical characteristics, comorbidity patterns, organ damage degrees and the potential for negative health outcomes, and this is particularly characteristic of elderly patients (65+), who, in turn, make up a major part of T2D patients [10,65]. This group experiences T2D alongside aging and the accumulation of comorbidities and geriatric syndromes such as sarcopenia, malnutrition, cognitive impairment and frailty, which can alter the pathophysiology of T2D, treatment effects and outcomes [10]. 

In particular, older T2D patients with frailty are more prone to hypoglycemia and its adverse consequences, including falls, fractures, hospitalization, CV events and mortality. Consideration should be given to simplification, switching or de-escalation of the therapeutic regimen in these patients [66,67]. However, if insufficiently treated, hyperglycemia can lead to acute complications such as dehydration, poor wound healing and hyperglycemic hyperosmolar coma, which should be avoided [67]. There is a delicate balance between over-treating and suboptimal treating, which requires an individualized treatment approach, carefully planning both pharmacological and non-pharmacological treatments [68]. Sometimes, it is not clear how much the under-prescription of the guideline-recommended therapy to frail patients, and how much the frailty status per se, contributes to poor outcomes [69].

**Figure 2:**
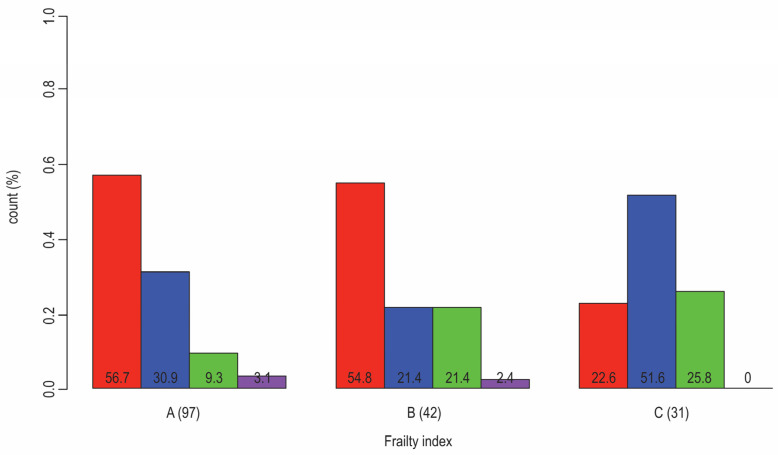
Distribution of patients that have T2D and are diagnosed with CVD (red: no CAD or CHD; blue: both CAD and CHD; green: only CHD; violet: only CAD) according to frailty status (A: nonfrail, B: pre-frail, C: frail); Fisher Test *p*-value (0.003). The results from our research (N = 170, F:M = 95:75, age 50–89 years, median 66). The results from our research that is cited in ref. [33].

Frailty is considered a state of failure of homeostasis in multiple organs and systems and is manifested by non-specific symptoms and signs such as muscle mass reduction, slow walking, low activity and a feeling of exhaustion, which are progressive in number and severity with aging and the presence of comorbidities [70]. Experts agree that an assessment of frailty should become a part of older T2D patients’ examination so that glycemic targets and therapeutic choices can be modified accordingly [67]. Nevertheless, knowledge is still insufficient to allow for the development of formal guidelines to help healthcare providers in their decisions on how to precisely manage these patients. One of the reasons may be the complex interplay between T2D, CVD, CKD, sarcopenia and frailty (Figure 2). These conditions share common pathophysiological pathways and can potentiate the development of each other [68,71,72]. 

Frailty is associated with incident T2D in an older population and an increased risk of comorbidities and poor outcomes; vice versa, T2D predicts the transition to higher frailty levels, while the vascular complications of T2D and associated malnutrition accelerate the functional decline associated with frailty [68]. 

**Figure 3:**
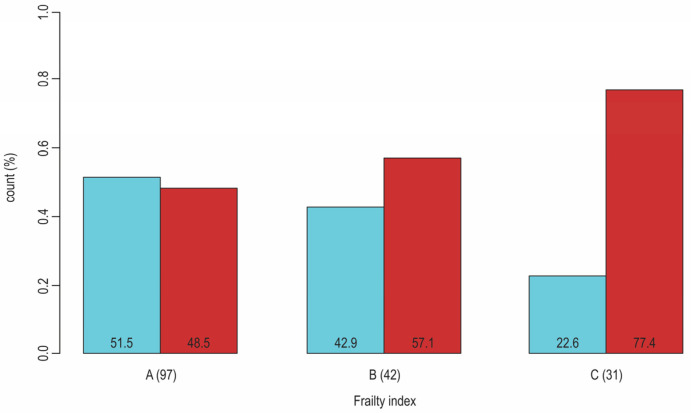
Sex-dependent distribution (blue: men, red: women) of patients with T2D according to their frailty status (A: nonfrail, B: pre-frail, C: frail); Pearson Chi-Square (*p*-value = 0.02). The results from our research (N = 170, F:M = 95:75, age 50–89 years, median 66). The results from our research that is cited in ref. [33].

Frailty contributes to the heterogeneity of patients with T2D. At least two frailty phenotypes exist in older T2D patients. One is associated with obesity and high insulin resistance (sarcopenic obese phenotype), and another is associated with weight loss, the body’s shrinking and low insulin resistance (anorexic malnourished phenotype) [73]. The growing evidence indicates that the clinical expression of frailty is sex-dependent, which means that women are more prone to frailty and frailty-related physical disability than men, while men experience frailty at older ages than women [74,75] (Figure 3). Contrary to what is the case in the general population, women with T2D are more prone than men with T2D to CVD, but preferably for a non-atherosclerotic type of CAD and CHD [76,77].

In summary, the relationship between T2D and frailty is complex, and many questions are still unanswered, which makes the treatment of older T2D patients challenging. This can also be applied to the treatment of these patients with new antidiabetic drugs, GLP-1ra and SGLT2-in (Table 3).

Although the common conclusion of the studies performed so far is that SGLT2-in and GLP-1ra improve CV outcomes in older (≥65) and frail patients, concerns remain when narrowly defined patient subgroups are used for the analysis such as older men and those older than 70 years [78]. In addition, frailty may change the harm–benefit balance of these drugs. One of the main concerns about their use in older or frail patients, particularly regarding GLP-1ra, is the effect of these drugs on weight loss, which, in these patients, could be counterproductive. For SGLT2-in, it is also important to take care of the presence of urinary incontinence, a disorder often associated with frailty, since the use of SGLT2-in may lead to the worsening of this disorder or cause serious infections in these patients [59]. In addition, the reduced hypoglycemic effect of SGLT2-in patients with low renal function may potentially increase the risk of diabetic ketoacidosis [59,67]. 

Although evidence exists concerning the effects of SGLT2-in regarding patients’ CV comorbidity severity stratification, such as the baseline patient stratification as to the presence of ASCVD, heart failure and degrees of renal function decline, it is not sufficient if we want to consider the magnitude of treatment effects, as well as the harm–benefit trade-off, in a more personalized context [84]. This is especially true for elderly patients with T2D, where many factors interact in predicting the measurable outcomes, of which some may have alleviating effects and some may have worsening effects. In our recently published article, we demonstrated that the level of inflammation may vary among T2D patients, which is determined at least by variables such as age, sex, BMI and the level of frailty [33]. Any level of frailty, including mild, moderate and severe, was shown to increase the risk for all-cause and CV-related mortality in patients with CKD, but with different magnitudes of influence [85]. The presence of frailty is likely to be a stronger predictor of CKD outcomes than the degree of renal function decline, measured by the estimated glomerular filtration rate (eGFR) [86]. 

Following an increased awareness of the heterogeneity of T2D patients and the requirement for individualized treatment, an initiative has been launched for precision medicine in diabetes [87,88]. Studies that assess the feasibility of using clustering techniques from data science application areas are underway, wanting to identify subcategories of patients with T2D that can be discriminated against in terms of CV and other poor outcomes and responses to treatment. Our research group has contributed to these efforts [32,33,89]. Regarding treatment, precision medicine looks at variations in drug effectiveness in specific patient subgroups and seeks markers (especially genetic markers) that can predict adverse drug events [87]. However, many challenges still need to be overcome before it will be possible to implement precision medicine in the management of T2D patients.

## 5. Discussion

Taken together, the evidence is still limited on how different patient features, including age, sex, body mass and shape, comorbidity patterns, frailty status and the level of renal function decline, may impact differences in how individuals respond to GLP-1ra and SGLT2-in [53,87,90]. This is partly due to the traditionally inadequate characterization of participants in clinical trials, who are not systematically assessed for comorbidities, functional status and frailty [53]. Clinical inertia is known to be exacerbated by ambiguous guidelines and pathways. On the other hand, being aware of the broader patient context, and how it may predict responses to certain treatments, will allow for better-informed decisions for the personalized management of patients with T2D [34]. 

According to the above discussion, the heterogeneity of older patients with T2D and an insufficient understanding of the factors that influence treatment outcomes in older patients with T2D might be key barriers to individualized patient care and reasons for the poor adherence to guidelines among healthcare providers and family physicians. For healthcare professionals to safely prescribe antidiabetic medications and make decisions about when to escalate or de-escalate treatment, the guidelines should be designed to assist the treatment of older adults with type 2 diabetes. This would be especially important for family physicians, who usually do not feel confident enough to radically change therapy by themselves. The international authorities emphasize the necessity of comprehensive patient assessment, which would allow for a multilayered and holistic approach to managing these patients [87]. Data indicating comorbidities, co-medications, functional disabilities, mental health disorders, doctor–patient communication, patient health literacy, issues such as a willingness to change or a preference for a certain type of therapy and the patient’s need for support are all to be taken into account. Many of these factors have an impact on medication uptake rates and, ultimately, on the patient response to the treatment and the outcomes. 

Evidence from epidemiologic research indicates that the time perspective of disease progression is critical to consider for hyperglycemia management, treatment regimen planning and the prediction of CV events in T2D patients. Variables such as T2D duration, patient age and age at T2D diagnosis prove prognostically meaningful, considering that a diagnosis earlier in the life course, at a younger age but with a longer T2D duration, leads to a higher CV risk [91]. The fact that CKD, which usually accompanies T2D, is regarded as an independent CV risk factor is also an important issue to take care of [92,93]. The variables specifically significant for the prognosis of T2D patients are age at T2D onset, eGFR and HbA1c. These variables were aligned with the latest classification system for CV event risk estimation (SCORE2), which comprises classical CV risk factors such as age, sex, smoking, systolic blood pressure and total and HDL-cholesterol, and re-calibrated into a new system, SCORE-2 Diabetes, used for estimating the ten-year risk of CV events in T2D patients of European countries [21,94]. 

By allowing T2D patients to be included in CV risk assessments as those who are most at risk for CVD, the model mentioned provides important advancements in the prevention of CVD [95]. Many CV risk prediction models applicable to patients with T2D have been developed so far, but they cannot accurately predict individuals who will probably experience CVD [96,97]. The applicability of SCORE-2 Diabetes has yet to be proved, concerning the accuracy of the prediction and the adequacy of the risk factors that have been included in the model [98]. An in-depth evaluation of T2D patients is still necessary to precisely identify individuals with subclinical CVD who are at a very high CV risk and are, therefore, also candidates for introducing therapy with new antidiabetic drugs [18,99]. Once new, efficacious cardiac biomarkers are approved for routine usage, routine testing using them will be a more straightforward method of accurately screening these patients [99,100]. 

Another problem is that the classical CV risk factors (which make up the SCORE2 model) do not perform as well as CV risk predictors in elderly individuals, while some new variables like pharmacologic treatments, cognitive decline and frailty have been proven to be better predictors. This makes the SCORE-2 Diabetes model particularly uncertain when it comes to predicting CV risk in elderly T2D patients [101,102]. There are also uncertainties related to the effect of sex on CV outcomes in T2D patients and the expression of frailty phenotypes, which may have implications for responses to treatment [76,103,104]. 

## 6. Future Directions

Precision medicine hoped to improve the health of individuals or specific population subgroups by identifying biomarkers (genetic, epigenetic or biochemical) for the early detection of important diseases which, in turn, would guide interventions [105]. The success of this approach has been shown partially. One of the primary causes is the potential for exceedingly complex disease etiology, particularly in the case of prevalent non-communicable diseases. In chronic complex diseases, genetic associations have a small effect size on the expression of phenotypes, in contrast to the more robust contribution of behavioral and social factors [87]. Moreover, these diseases develop as a part of the aging process, by sharing common pathophysiological pathways with aging and with each other, showing a tendency to cluster together [106]. Knowing the clinical, biological and sociodemographic characteristics that are consistently linked to variations in clinical outcomes is essential for treating patients with chronic complicated diseases on an individual basis [53]. 

Today, there is an emerging trend in using large-scale person-generated health data from electronic health records, smartphones and wearables to characterize different patient subgroups and to improve the health and well-being of particular patient subgroups through strategies customized to their specific characteristics [107]. Based on our own experience, we recommend the implementation of Artificial Intelligence (AI) and data-driven research methods in primary care and family medicine to become a part of the routine healthcare workflows [108]. As an answer to doubts about the accuracy and repeatability of the results of these methods, it is worth mentioning that the techniques in the field of AI applications that already exist can guarantee the generalizability of findings or can consider the patient effect heterogeneity. It might diminish uncertainties associated with patient complexity and support family physicians in more individualized decisions, especially in areas where guidelines cannot provide clear recommendations. The search for simple-to-obtain biomarkers of CVD or frailty that can be used in population-based studies could also help harness uncertainty. 

In addition to CVOTs, further research efforts should focus on preparing real-life studies, aimed at addressing complex issues such as different comorbidity patterns. The findings from clinical trials cannot be generalized to the population at large due to the stringent eligibility criteria. Studies based on “real-world data” are increasingly used to complement clinical trials. Furthermore, these studies can provide information that is not possible to obtain by clinical trials, such as natural history and the course of disease, effectiveness studies, outcome studies and safety surveillance. However, they have some important limitations. Unlike randomized trials, in observational studies, the treatment is not actively assigned for research purposes but is based on subject characteristics. This may result in incorrect (biased) estimations of the treatment effect because the treated and the control group may have large differences in their characteristics (covariates). Thus, a major challenge in “real-world” studies is how to balance the patient characteristics contained in electronic health records and other sources with routinely collected data for the given measure of treatment effectiveness. The propensity score (PS) method estimates the likelihood of being treated given covariates and is emerging as a confounding adjustment method. When estimated, PS can be used to reduce bias through methods such as matching, weighting, stratification (subclassification), regression adjustment or a combination of methods. The PS accounts only for observed confounders. Variable selection for inclusion in a PS model can range from narrowly selected covariates, based on expert choice or existing knowledge, to a large number of empirically selected covariates that require the use of data-driven methods for data preprocessing and optimization [109,110]. 

The great problem in validating the model’s predictive performance appears when some latent confounders, which are not captured by the PS, because they are either not recorded in the database or not recognized as important, significantly influence the treatment effect, leading to biased estimates and, thus, to wrong conclusions. These are situations that include selection bias, which occurs when the selection of the participants or follow-up time is related to both the interventions and the outcomes. In the former case, an example is when the clinician’s decision to treat a patient involves the severity of the condition, which has not been measured (“confounding by indication”), or when some individuals receive the treatment depending on their characteristics such as socioeconomic status or a health-related behavior, characterized by a good adherence to treatment. Overcoming this problem requires sensitive analytical procedures, the use of some additional, still poorly validated computer methods or the confirmation of the treatment effect through performing randomized control trials [111].

The inappropriate accounting of follow-up time and treatment status in the design and analysis of the cohort studies can introduce immortal time bias, which could, e.g., contribute to our misunderstanding of the benefits of treatment with new antidiabetic drugs, compared to classical oral antidiabetic drugs or other concomitant medications [112]. Several approaches have been proposed to prevent immortal time biases that are based on the cautious preparation of the study design, in addition to the switch from a time-fixed to a time-dependent analysis. 

To promote the routine use of “real-world data” in clinical research and to speed up the filling of the evidence gaps, these studies need to be performed transparently and with integrity, use fit-for-purpose data and address the key risks of bias [113].

To summarize, the unresolved issue of how to manage the heterogeneity of patients with T2D and to define subgroups with different levels of CV risk is the main barrier to individualized treatment and the reason for the low uptake of GLP-1ra and SGLT2-in drugs, despite accumulating evidence on their CV benefits and decreasing costs. The shortcomings of the guidelines primarily reflect the methodological limitations of the current evidence base. The intensification of research, with the introduction of new research methods and approaches, is necessary to fill the current research gaps and allow for the translation of new evidence into the guidelines’ recommendations and clinical practice. Priority should also be given to advancing translational and implementation sciences, which should obligatorily include qualitative research including primary care providers and family physicians. This will hopefully help remove obstacles to the practical application of the guidelines in practice and tailored recommendations. Further research efforts should ultimately involve the discovery of new biomarkers for CVD and frailty. 

We summarized priorities for future work that should fill the gaps in the current evidence-based recommendations, as identified by this review, including strategies that refer to designing future research, the process of the guidelines’ development and knowledge implementation strategies (Table 4). 

## 7. Conclusions

Current evidence on the treatment effect heterogeneity for GLP1-ra and SGLT2-in therapies is limited, reflecting the methodological limitations of the underlying research. The introduction of new research methods and approaches is necessary to fill the current research gaps and allow for an understanding of treatment effect heterogeneity in T2D patients. The translation of new evidence into the guidelines’ recommendations and clinical practice needs to involve different methods and more active approaches. Clear and unconditional recommendations for the individualized management of patients with T2D may encourage the prescription of these drugs by the providers, which is especially crucial for family physicians who deal with a wide range of specific patient contexts daily, as well as various clinical and social settings. 

## Figures and Tables

**Table 1:** Factors influencing therapeutic/clinical inertia.

Physician-Related Factors:	Patient-Related Factors:	Healthcare-Related Factors:
Lack of knowledge about the disease Overlooking the seriousness of the symptoms Not starting the treatment Inability to identify clinical outcomes Not adjusting the treatment until the intended result has been attained Inability to recognize and address comorbid conditions An assumption that a patient will not follow recommendations to intensify the therapy Lack of knowledge about the guidelines Reactive care as opposed to a proactive treatment strategy	Refusal to accept having the illness Non-existence of symptoms Inadequate knowledge of the health Considering that the illness is not severe Taking too many medications Financial limitation (cost of medications) The negative effects of medications Aspects of lifestyle Ineffective patient–doctor communication (lack of trust)	The absence of clinical guidelines No database of morbidity or disease registries No visit strategy (not scheduling appointments) The absence of decision assistance and team-based care Insufficient funds and time Technical deficiency

**Table 2:** Possible side effects and heterogeneous treatment effects for SGLT2-in and GLP-1ra (according to refs. [53,58,59,60]).

Heterogeneous Treatment Effects	Possible Side Effects
Higher baseline HbA1c values are associated with greater glycemic responses. GLP-1ra: There is no proof that renal function affects the response to glucose. SGLT2-in: Renal function needs to be regularly monitored due to a possible reduction in GFR. There is not any reliable proof that one’s body mass index significantly modifies the glycemic response. GLP-1ra: Less of a glycemic response is linked to a longer duration of diabetes. SGLT2-in: The length of diabetes does not consistently affect the glycemic response. GLP-1ra: No proof that aging affects the glycemic response. SGLT2-in: Elderly patients and those with renal impairment should use much more caution. No reliable proof that gender or ethnicity significantly modifies the glycemic response. SGLT2-in: No indication of variations in the reaction to treatment for patients with obstructive sleep apnea or insulin secretion and insulin. GLP-1ra: Studies indicate that lower fasting C-peptide- and urine C-peptide-to-creatinine ratios are linked to a reduced glycemic response.	Concerns regarding the start of SGLT2-in in patients with CVD or kidney illness as well as the necessity of the close observation of diabetic ketoacidosis (DKA) in cases of COVID-19 or other acute conditions requiring hospitalization. There is a higher risk of genital and urinary tract infections, which can be attributed to prolonged and increased glycosuria. Raised concerns about gliflozin-induced cancers (bladder and breast cancer), even though causality cannot be established. More research is required. Gliflozins cannot rule out drug-induced liver damage. Due to SGLT1 transporters’ reduced inhibition, there may be a lower risk of severe hypoglycemia when using gliflozins. Hypovolemic events are frequent with gliflozins, and DKA can appear infrequently and be more challenging to diagnose. Gliflozins-associated fracture risk is likely influenced by several variables that require additional research. Renal function needs to be regularly monitored due to a possible reduction in GFR. Elderly patients and those with renal impairment should use much more caution. Although evidence indicates that SGLT2 inhibition increases total serum cholesterol, this effect relates to a greater rise in the less atherogenic LDL-cholesterol subfraction. In addition, the ratio of LDL- to HDL-cholesterol remains nearly unaltered because of a concurrent rise in HDL-cholesterol.

HbA1c: hemoglobin A1c; GLP-1ra: glucagon-like peptide 1 receptor agonists; SGLT-2in: sodium-glucose cotransporter-2 inhibitors; CVD: cardiovascular disease; DKA: diabetic ketoacidosis; LDL: low-density lipoprotein; HDL: high-density lipoprotein.

**Table 3:** A list of studies indicating the effect of older age and/or frailty on treatment effects with GLP-1ra and SGLT-2-in in patients with T2D.

Study (Design)	Findings Summary
Lin TK, et al., 2023. (population-based longitudinal cohort study) ref. [78]	SGLT2-in use was associated with a non-significantly decreased risk of ACS. No difference in the SGLT2-in subtype was observed in subgroup analyses. The results indicated an increased risk for the incidence of ACS in male and older (>70 years) patients.
Leong DP, et al., 2023. (population-based longitudinal cohort study) ref. [79]	Frailty confers substantial incremental prognostic information to prognostic variables for predicting death and hospitalization in heart failure patients. The relationship between frailty and these outcomes is consistent across countries at all income levels.
Kutz A, et al., 2023. (1:1 propensity score-matched cohort studies) ref. [80]	SGLT2-in and GLP-1ra safely improved CV outcomes and all-cause mortality, with the largest absolute benefits among frail people.
Young KG, et al., 2023. (a systematic review) ref. [53]	Current evidence on treatment effect heterogeneity for SGLT2-in and GLP-1ra therapies is limited, likely reflecting the methodological limitations of published studies. Robust and appropriately powered studies are required to understand T2D treatment effect heterogeneity.
Strain WD, Griffiths J, 2021. (a systematic review and meta-analysis) ref. [81]	GLP-1ra and SGLT2-in reduced MACE outcomes in older adults who were eligible to participate in clinical trials. Whereas this is reassuring for the biologically robust, it should not be extrapolated to frail older adults without further investigation.
Karagiannis T, et al., 2021. (a systematic review and meta-analysis) ref. [82] Vart P, et al., 2023. (a randomized controlled trial) ref. [83]	In older adults (≥65) with T2D, GLP-1ra reduced MACE and its components. SGLT2-in reduced MACE, heart failure and renal outcomes. The relative benefit of dapagliflozin in patients with chronic kidney disease (with/without T2D) for all outcomes was consistent across all frailty categories, with no difference in associated safety.

ACS: acute coronary syndrome; MACE: major cardiovascular event; T2D: type 2 diabetes; CV: cardiovascular; HF: heart failure.

**Table 4:** Future directions for overcoming T2D patient heterogeneity and increasing the uptake of GLP-1ra and SGLT2-in drugs.

Research	Guidelines Development	Knowledge Implementation
More comprehensive patient profiling for CVOTs Real-world data-driven research Patient clustering based on multiple descriptors (including comorbidity patterns) Research intensification on the treatment effects of non-pharmacological interventions, particularly including psychological factors New biomarkers exploration Integrated models of care Qualitative research—assessing family doctors’ attitudes Translation research intensification	Clear division of areas where there is no support by evidence Case report Examples dealing with real-life experience Monitoring for the side effects of the treatment regimens The effects of comorbidity and frailty on treatment outcomes Evidence provided by cluster-analyses Clear recommendations on when to intensify and when to de-escalate therapy Clear statements on when to start with the cardio-protective therapy in the course of the disease The effects on outcomes of non-pharmacological interventions, particularly concerning psychological factors Problem-solving approach Guidelines adaptation for use in primary care	Continuous knowledge translation Educational training modules for healthcare providers, health policy officials and patients Learning on case reports Audit analysis of real-world data with the support of information technology Implementation of care protocols and workflows Empowerment of family doctors in life science knowledge The establishment of the platform for data collection and curation from electronic health records, smartphones and wearables Data selection for data-driven modeling The results of data modeling become visible in the workplace by using AI visual techniques

## Data Availability

The data presented in this study are available on request from the corresponding author.

## Abbreviations

T2D	type 2 diabetes
CV	cardiovascular
GLP-1ra	glucagon-like peptide 1 receptor agonists
SGLT-2in	sodium-glucose cotransporter-2 inhibitors
CVD	cardiovascular disease
ADA	American Diabetes Association
EASD	European Association for the Study of Diabetes
HbA1c	hemoglobin A1c
LDL	low-density lipoprotein
HDL	high-density lipoprotein
CAD	coronary artery disease
ASCVD	atherosclerotic cardiovascular disease
CVOT’s	cardiovascular outcome trials
CHF	chronic heart failure
CKD	chronic kidney disease
AI	Artificial Intelligence
DKA	diabetic ketoacidosis
PS	propensity score
